# Porcine single nucleotide polymorphisms and their functional effect: an update

**DOI:** 10.1186/s13104-018-3973-6

**Published:** 2018-12-04

**Authors:** B. N. Keel, D. J. Nonneman, A. K. Lindholm-Perry, W. T. Oliver, G. A. Rohrer

**Affiliations:** USDA, ARS, U.S. Meat Animal Research Center, Clay Center, NE 68933 USA

**Keywords:** Swine, Genome sequence, Functional variation, Loss-of-function

## Abstract

**Objective:**

To aid in the development of a comprehensive list of functional variants in the swine genome, single nucleotide polymorphisms (SNP) were identified from whole genome sequence of 240 pigs. Interim data from 72 animals in this study was published in 2017. This communication extends our previous work not only by utilizing genomic sequence from additional animals, but also by the use of the newly released Sscrofa 11.1 reference genome.

**Results:**

A total of 26,850,263 high confidence SNP were identified, including 19,015,267 reported in our previously published results. Variation was detected in the coding sequence or untranslated regions (UTR) of 78% of the genes in the porcine genome: 1729 loss-of-function variants were predicted in 1162 genes, 12,686 genes contained 64,232 nonsynonymous variants, 250,403 variants were present in UTR of 15,739 genes, and 15,284 genes contained 90,939 synonymous variants. In total, approximately 316,000 SNP were classified as being of high to moderate impact (i.e. loss-of-function, nonsynonymous, or regulatory). These high to moderate impact SNP will be the focus of future genome-wide association studies.

**Electronic supplementary material:**

The online version of this article (10.1186/s13104-018-3973-6) contains supplementary material, which is available to authorized users.

## Introduction

One of the key aims of livestock genomics research is to identify genetic variation underlying economically important traits such as reproductive performance, feed efficiency, disease resistance/susceptibility, and product quality. Until recently, association studies and genomic predictions have been performed using commercial single nucleotide polymorphism (SNP) arrays, which contain markers evenly spaced across the genome. Accuracy of genomic predictions can be improved by using more informative variants, including variants located near or within genes, predicted to affect gene function, or known to be causal. Genetic variants detected from whole-genome sequence have been used to successfully identify causal variants and to map complex traits in domestic cattle [1–4]. In particular, it was shown that loss-of-function (LOF) variants, those expected to disrupt the protein coded by a gene, in the homozygous state can compromise fertility in cattle by causing embryonic lethality [1]. Hence, a comprehensive list of LOF variants, as well as variants that alter the amino acid structure of a protein and those that regulate protein production, detected from whole-genome sequencing would be of considerable interest in swine genomic studies, particularly those targeting fertility and production traits.

The porcine variation currently reported in National Center for Biotechnology Information (NCBI) genetic variation database (dbSNP) and the European Variation Archive (EVA) represents several diverse pig breeds and wild boars from different regions of the world. Therefore it is likely that many of the variants in these databases do not segregate in commercial swine germplasm, a consequence of domestication and selection for lean meat production. To provide information on variants predicted to affect gene function in commercial swine germplasm, a study was conducted to identify single nucleotide polymorphisms (SNP) from whole-genome sequence of 240 members of an experimental swine herd at the U.S. Meat Animal Research Center (USMARC). These animals included all 24 of the founding boars (12 Duroc and 12 Landrace), 48 of the founding Yorkshire-Landrace composite sows, 109 composite animals from generations 4 through 9, 29 composite animals from generation 15, and 30 purebred industry boars (15 Landrace and 15 Yorkshire) used as sires in generations 10 through 15. Interim results from the 72 founding animals have been previously published [5]. The objective of this report is to present the updated data following sequencing of an additional 168 animals and the release of the new and improved swine reference genome build, Sscrofa 11.1.

## Main text

### Materials and methods

The DNA samples sequenced for this study were extracted from semen collected by commercial artificial insemination services and from blood and tail tissue archived under standard operating procedures for the USMARC tissue repository. The research did not involve experimentation on animals requiring Institutional Animal Care and Use Committee approval.

#### Library preparation and sequencing

Blood, semen, or tail tissue samples were obtained from 240 members of a USMARC composite population, 72 founding animals (generation 0), 109 animals from generations 4 through 9, 29 animals from generation 15, and 30 industry sires. DNA extraction, library preparation, and sequencing for the 72 founding animals has been previously described in [5]. For the 36 animals in generations 4 and 5, genomic DNA was extracted from tail tissue using standard DNA extraction protocols, sheared to 300–500 bp using a Covaris S220 ultrasonicator (Woburn, MA, USA), and libraries prepared using the TruSeq DNA sample prep kit, version 2 (Illumina, San Diego, CA, USA) were paired-end sequenced (100 bp read length) on an Illumina HiSeq 2500 (Illumina Inc., San Diego, CA, USA) at DNA LandMarks (St.-Jean-sur-Richelieu, QC, Canada). Genomic DNA for the 30 AI sires, the 73 animals from generations 6 through 9, and the 29 animals from generation 15 was extracted using a Wizard SV96 genomic DNA purification kit according to the manufacturer’s protocol (Promega Corp., Madison, WI, USA). Genomic DNA was sheared to 350 bp (generation 15 animals) or 550 bp (AI sires and generation 6 through 9 animals) using a Covaris S220 ultrasonicator (Woburn, MA, USA), and libraries prepared using the TruSeq DNA PCR-Free prep kit (Illumina, San Diego, CA, USA). Libraries were paired-end sequenced (150 bp read length) on an Illumina NextSeq 500 (Illumina, San Diego, CA, USA) at USMARC. Bases of the paired-end reads for all sequenced genomes were identified with the Illumina BaseCaller, and FASTQ files were produced for downstream analysis of the sequence data.

#### Sequence data processing

The Trimmomatic software (Version 0.35) [6] was used to trim Illumina adaptor sequences and low quality bases from the reads. The remaining reads were mapped to the Sscrofa 11.1 genome assembly (NCBI Accession AEMK00000000.2) using Burrows–Wheeler Alignment (BWA, Version 0.7.12) [7] with the default parameters. All output SAM files were converted to sorted BAM files using SortSam from Picard (Version 1.1; http://broadinstitute.github.io/picard/), and duplicates in the BAM files were marked by applying MarkDuplicates from Picard. Genomic coverage for each of the BAM files was computed using Samtools (Version 1.3) [8].

#### Variant calling and filtering

Best practices established for the Genome Analysis Toolkit (GATK, Version 3.7) [9] were used to identify SNP. Briefly, RealignerTargetCreator and IndelRealigner from GATK were applied for local realignment of indels. Base quality recalibration was then performed using BaseRecalibrator from GATK, where the recalibration report was formed using the default setting for covariates and the NCBI dbSNP database (Build 150) as the database for known sites.

Multi-sample variant calling and genotyping was performed with the GATK UnifiedGenotyper, taking input from each of the 240 BAM files.

The UnifiedGenotyper output provides several metrics that assess the quality of detected variation, including quality (QUAL), quality by depth (QD), RMS mapping quality (MQ), Fisher strand (FS), haplotype score (HaplotypeScore), mapping quality rank sum test (MQRankSum), and read position rank sum test (ReadPosRankSum). The QUAL metric is a phred-scaled probability of the SNP being homozygous for the reference, where higher values indicate higher confidence. QD is computed by dividing the variant confidence (QUAL) by the unfiltered depth of all non-reference samples. FS is a phred-scaled P-value using Fisher’s Exact Test to identify strand bias. Higher FS values indicate stronger strand bias, i.e. likely false positives. The HaplotypeScore is a measure of how well the data in a 10-base window around the variant can be explained by at most 2 haplotypes. MQRankSum is a Wilcoxon Rank Sum Test that tests the hypothesis that the reads carrying the variant allele have a consistently lower mapping quality than the reads with the reference allele, while ReadPosRankSum is a Mann–Whitney Rank Sum Test that tests the hypothesis that instead of being randomly distributed over the read, the variant allele is consistently found more often at the beginning or the end of a sequencing read. In order to reduce the false discovery rate, variants were hard filtered to meet the following criteria, suggested by GATK documentation: QUAL > 30.0, QD > 2.0, MQ > 40.0, FS < 60.0, HaplotypeScore < 13.0, MQRankSum > − 12.5, and ReadPosRankSum > − 8.0.

#### Classification of variants

Filtered variants were classified according to their expected effect on gene function using snpEff (Version 4.3) [10] and NCBI (Release 106) annotation of the Sscrofa 11.1 build. Variants detected in coding sequence were classified into one of four functional categories: (1) loss-of-function SNP, which are high impact variants expected to disrupt the protein coded by the gene; (2) non-synonymous SNP, which are moderate impact variants that alter the amino acid sequence of the protein coded by the gene, (3) regulatory SNP, which occur in non-coding RNA and untranslated regions (UTR) of protein-coding genes; and (4) silent SNP, which are synonymous SNP and other low impact variants that do not affect the amino acid sequence.

#### Function of genes containing variation

Functions of genes containing detected variants were determined using the PANTHER classification system (Version 13.0) [11]. Enrichment analysis of gene function was performed using PANTHER’s implementation of the binomial test of overrepresentation. Significance of gene ontology (GO) terms was assessed using the default *Sus scrofa* GO annotation as background for the enrichment analysis. Data were considered statistically significant at Bonferroni corrected *P*-value < 0.05.

### Results and discussion

Genomic DNA from 240 pigs, from a composite population at USMARC, was sequenced on the Illumina HiSeq and NextSeq platforms, generating approximately 72 billion paired-end reads (Additional file 1: Table S1). Sequence reads covered each pig’s genome at a mean of 13.62 fold (×) coverage. Individual coverage per animal ranged from 0.97× to 31.13×; 24 animals were covered at less than 3×, and 44 were covered at more than 20×.

A total of 26,850,263 high confidence SNP were identified. Our variants comprised 36.9% of the porcine SNP in the NCBI dbSNP database (Build 150). A total of 5793,049 of our variants were novel, i.e. not present in dbSNP, and 45,411 of them overlapped with the 61,596 SNP assayed by the PorcineSNP60 v2 BeadChip (Illumina Inc., San Diego, CA). While approximately 94% of the variants from our previous report were also identified in this study, only 85% of variants from our previous study were identified as high confidence variants (passing quality filtering). One factor contributing to this discrepancy was the use of the improved reference genome, where many of the gaps and misassemblies present in the Sscrofa 10.2 genome build were resolved. Furthermore, the addition of high coverage sequence for 168 animals improved the allele frequency calculations used during multi-sample variant calling by GATK’s UnifiedGenotyper software, thereby producing quality scores that are much more refined than those from our previous study.

The number of SNP detected in our pigs was similar to previous reports [12, 13]. The variants reported in dbSNP represent several diverse pig breeds and wild boars from different regions of the world. Therefore the detection of less than half of dbSNP’s variants in our animals is likely because those variants do not segregate in our population, which represents commercial swine germplasm, as a consequence of domestication and selection for lean meat production.

**Fig. 1:**
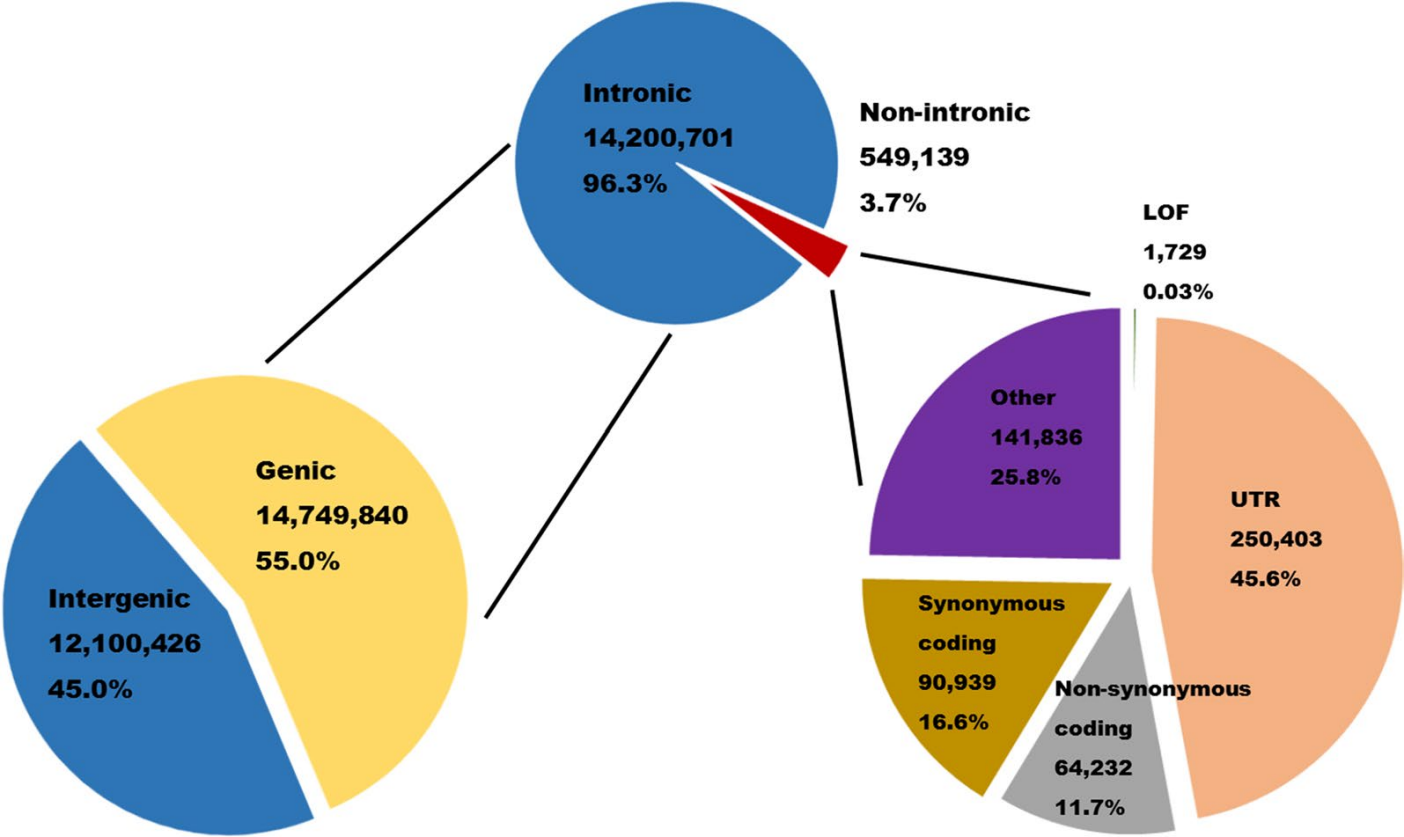
Overview of the 26,850,263 SNP identified. Here, variant annotations have been collapsed so that each variant has only a single annotation. The category “Other” includes variants downstream of genes (up to 5 Kb), variants located in splice sites, and variants present in a gene but not in any of its transcripts

Variation was detected in 23,272 of the 29,847 annotated porcine genes (Additional file 2: Table S2). Most variation was detected in intergenic and intronic regions (Fig. 1). Analysis of expected effect on gene function predicted that 1162 protein-coding genes may be affected by 1729 loss-of-function or other high impact variants, 12,686 genes contained 64,232 moderate impact variants (nonsynonymous and other SNP altering the coded amino acid sequence), and 15,284 genes contained 90,939 low impact variants (SNP not expected to alter the amino acid sequence). Additionally, 250,403 variants that may be regulatory were identified, located in untranslated regions (UTR) of 15,739 genes.

Genes involved in hydrolase activity, ion binding, and nucleotide binding were among those over-represented in the set of genes containing high impact variants (Table 1). Large numbers of genes in the nonsynonymous, synonymous, and regulatory classes resulted in large numbers of significantly over-represented GO terms. The most significant molecular function GO terms that were over-represented in genes with nonsynonymous variants were related to protein and ion binding (Additional file 3: Table S3). Similarly, GO terms related to protein binding, ion binding, and nucleic acid binding were among the top over-represented terms in the set of genes with synonymous and regulatory variants (Additional file 4: Tables S4, Additional file 5: Table S5).

**Table 1:** Significantly over- and underrepresented gene ontology (GO) terms in protein-coding genes with loss-of-function variants

Ontology term	Gene set (n genes)		LOF genes expected	Over (+) or under (−)	P-value^a^
	Annotated genes (22,191)	LOF genes (592)			
Biological process					
Meiotic cell cycle (GO: 0051321)	103	13	2.87	+	4.38E−02
Cellular process (GO:0009987)	8666	319	241.73	+	3.26E−06
Localization (GO: 0051179)	2853	123	79.58	+	5.12E−03
Molecular function					
Phospholipid-translocating ATPase activity (GO:0004012)	15	5	0.42	+	2.48E−02
Intracellular ligand-gated ion channel activity (GO:0005217)	17	5	0.47	+	3.20E−02
ATPase activity, coupled (GO:0042623)	159	16	4.44	+	4.31E−03
ATPase activity (GO:0016887)	205	17	5.72	+	2.16E−02
Calcium ion binding (GO:0005509)	483	29	13.47	+	3.35E−02
Pyrophosphatase activity (GO:0016462)	512	30	14.28	+	3.29E−02
ATP binding (GO:0005524)	1080	63	30.13	+	5.98E−05
Hydrolase activity, acting on acid anhydrides, in phosphorus-containing anhydrides (GO:0016818)	515	30	14.37	+	3.27E−02
Hydrolase activity, acting on acid anhydrides (GO:0016817)	516	30	14.39	+	3.13E−02
Adenyl nucleotide binding (GO:0030554)	1121	64	31.27	+	8.65E−05
Drug binding (GO:0008144)	1219	69	34	+	5.16E−05
Adenyl ribonucleotide binding (GO:0032559)	1115	63	31.1	+	9.88E−05
Carbohydrate derivative binding (GO:0097367)	1569	84	43.77	+	2.92E−05
Purine nucleotide binding (GO:0017076)	1391	74	38.8	+	1.09E−04
Purine ribonucleoside triphosphate binding (GO:0035639)	1344	71	37.49	+	1.89E−04
Purine ribonucleotide binding (GO:0032555)	1385	73	38.63	+	1.24E−04
Ribonucleotide binding (GO:0032553)	1398	73	39	+	1.82E−04
Nucleotide binding (GO:0000166)	1565	76	43.65	+	1.26E−03
Nucleoside phosphate binding (GO:1901265)	1565	76	43.65	+	1.19E−03
Small molecule binding (GO:0036094)	1801	87	50.24	+	2.89E−04
Anion binding (GO:0043168)	1927	93	53.75	+	1.33E−04
Hydrolase activity (GO:0016787)	1605	76	44.77	+	2.04E−03
Metal ion binding (GO:0046872)	2142	90	59.75	+	2.43E−02
Ion binding (GO:0043167)	3701	155	103.24	+	9.19E−05
Cation binding (GO:0043169)	2186	91	60.98	+	2.57E−02
Catalytic activity (GO:0003824)	3731	148	104.07	+	2.03E−03
Organic cyclic compound binding (GO:0097159)	3718	140	103.71	+	3.02E−02
Molecular_function (GO:0003674)	10,828	398	302.04	+	4.04E−11
Binding (GO:0005488)	7828	287	218.36	+	2.47E−05
Cellular component					
Integral component of membrane (GO: 0016021)	4022	151	112.19	+	3.21E−02
Intrinsic component of membrane (GO: 0031224)	4068	152	113.47	+	3.60E−02
Membrane (GO: 0016020)	5874	210	163.85	+	1.95E−02
Cell part (GO: 0044464)	9798	330	273.31	+	3.10E−03
Cell (GO: 0005623)	9847	332	274.67	+	3.43E−03

^a^Bonferroni corrected *P*-value

### Conclusion

Utilizing functional SNP could significantly boost the reliability of genomic predictions. Loss-of-function variants and others that disrupt or alter proteins coded by a gene, as well as those that regulate protein production, likely have a greater effect on phenotype than other types of variation. In this work, we identified 26,850,263 SNP, of which 316,364 were predicted to be of high to moderate impact. The present results provide an updated resource for functional variation in commercial swine germplasm.

## Limitations

This work is only the first step in identifying functional genetic markers that influence economically relevant traits in the US swine industry. Additional work, including imputation of sequence variants throughout our population and developing assays to directly genotype functional variants, is needed to discover the extent to which these variants affect specific traits of interest. Continued examination of the variants identified in this work is expected to lead to the development of genotyping panels that will allow swine producers and breeders to be able to make more rapid genetic progress by including them into their selection decisions.

## Additional files

**Additional file 1: Table S1.** Animals used in this study and summary of sequencing.

**Additional file 2: Table S2.** Genes containing loss-of function (LOF), nonsynonymous (NONSYN), synonymous (SYN), regulatory (UTR), and other variants and the positions of variants.

**Additional file 3: Table S3.** Significantly enriched gene ontology (GO) terms in the set of genes containing nonsynonymous variants.

**Additional file 4: Table S4.** Significantly enriched gene ontology (GO) terms in the set of genes containing synonymous variants.

**Additional file 5: Table S5.** Significantly enriched gene ontology (GO) terms in the set of genes containing regulatory variants.

## Abbreviations

SNP: single nucleotide polymorphism
UTR: untranslated region
LOF: loss of function
NCBI: National Center for Biotechnology Information
dbSNP: genetic variation database
EVA: European Variation Archive
USMARC: US Meat Animal Research Center
DNA: deoxyribonucleic acid
BWA: Burrows–Wheeler alignment
GATK: genome analysis toolkit
PANTHER: protein analysis through evolutionary relationships
GO: gene ontology
